# Best practice for motor imagery: a systematic literature review on motor imagery training elements in five different disciplines

**DOI:** 10.1186/1741-7015-9-75

**Published:** 2011-06-17

**Authors:** Corina Schuster, Roger Hilfiker, Oliver Amft, Anne Scheidhauer, Brian Andrews, Jenny Butler, Udo Kischka, Thierry Ettlin

**Affiliations:** 1 Reha Rheinfelden, Rheinfelden, Switzerland; 2 School of Health and Social Care, Oxford Brookes University, Oxford, UK; 3 Department of Health & Social Work, HES-SO, University of Applied Sciences, Western Switzerland, Sion, Switzerland; 4 Faculty of Electrical Engineering, Technical University Eindhoven, Eindhoven, The Netherlands; 5 Department of Information Technology and Electrical Engineering, ETH Zurich, Zurich, Switzerland; 6 Brunel Institute for Bioengineering, Brunel University, London, UK; 7 Nuffield Department of Surgical Sciences, Oxford University, Oxford, UK; 8 Oxford Centre for Enablement, Oxford, UK; 9 Department of Behavioural Neurology, Medical Faculty, University of Basel, Basel, Switzerland

## Abstract

**Background:**

The literature suggests a beneficial effect of motor imagery (MI) if combined with physical practice, but detailed descriptions of MI training session (MITS) elements and temporal parameters are lacking. The aim of this review was to identify the characteristics of a successful MITS and compare these for different disciplines, MI session types, task focus, age, gender and MI modification during intervention.

**Methods:**

An extended systematic literature search using 24 databases was performed for five disciplines: Education, Medicine, Music, Psychology and Sports. References that described an MI intervention that focused on motor skills, performance or strength improvement were included. Information describing 17 MITS elements was extracted based on the PETTLEP (physical, environment, timing, task, learning, emotion, perspective) approach. Seven elements describing the MITS temporal parameters were calculated: study duration, intervention duration, MITS duration, total MITS count, MITS per week, MI trials per MITS and total MI training time.

**Results:**

Both independent reviewers found 96% congruity, which was tested on a random sample of 20% of all references. After selection, 133 studies reporting 141 MI interventions were included. The locations of the MITS and position of the participants during MI were task-specific. Participants received acoustic detailed MI instructions, which were mostly standardised and live. During MI practice, participants kept their eyes closed. MI training was performed from an internal perspective with a kinaesthetic mode. Changes in MI content, duration and dosage were reported in 31 MI interventions. Familiarisation sessions before the start of the MI intervention were mentioned in 17 reports. MI interventions focused with decreasing relevance on motor-, cognitive- and strength-focused tasks. Average study intervention lasted 34 days, with participants practicing MI on average three times per week for 17 minutes, with 34 MI trials. Average total MI time was 178 minutes including 13 MITS. Reporting rate varied between 25.5% and 95.5%.

**Conclusions:**

MITS elements of successful interventions were individual, supervised and non-directed sessions, added after physical practice. Successful design characteristics were dominant in the Psychology literature, in interventions focusing on motor and strength-related tasks, in interventions with participants aged 20 to 29 years old, and in MI interventions including participants of both genders. Systematic searching of the MI literature was constrained by the lack of a defined MeSH term.

## Introduction

In sports psychology, there is evidence that mental practice (MP) can accelerate learning and improve motor skills. In their extensive meta-analysis in 1983, Feltz and Landers included single-group interventions with pre- and post-tests (tests before and after the interventions), and studies with multiple groups to compare an MP group versus controls [1]. They summarised 60 studies regardless of their quality and methods. Analysis of effect sizes showed that performing MP is not as good as physical practice (PP) but is better than doing no practice at all. In their revised meta-analysis in 1988, they replicated the previous results [2].

MP can be considered an umbrella term that includes various mental training interventions. In recent years, researchers have started to use the term 'motor imagery' (MI) to specifically address the imagination of moving specific body parts.

Over the past two decades, the publication of MP literature has increased tremendously, from 122 publications up to 1980 to a total of 20,011 publications in 2009 (PubMed search on 12 April 2010 with the search term 'mental practice'). The MI technique has been adopted in other research areas (education, medicine, music and sports), where the beneficial effect of MI added to PP has been confirmed, and 27 reviews summarise the research findings in those fields [1-27]. Despite the different review foci (for example, history and development of MI, theoretical concepts of MI functioning and effectiveness evaluation), all reviews attribute a beneficial effect to MI when added to PP. In some reviews, the methodological procedure lacked a systematic approach.

### Aim of the current systematic literature review

None of the published reviews have analysed the design of the MI training session (MITS) to determine successful MI intervention techniques, such as the position of the person during MI, the number of MI trials, and the instruction mode and type. However, the MITS design is of vital importance for researchers and clinicians planning to implement MI interventions adapted to participant health status, age and gender. In this systematic literature review, we extracted and analysed 17 MITS elements based on the PETTLEP (physical, environment, timing, task, learning, emotion, perspective) approach. Furthermore, we analysed seven temporal parameters, including duration times and number of repetitions. In total, we analysed five disciplines in which MI represents an important training strategy.

### Imagery models and frameworks in the education and psychology literature

Hall described the cognitive processes and neural basis of MI in a review on educational literature, based on a MEDLINE search [7]., and proposed a six-stage procedure for explicit learning of surgical skills: task definition, prior learning, mental rehearsal, reflection, problem solving and reality check.

In psychology, various tasks, participant groups and reporting statistics have been considered for MI. Driskell *et al*. summarised the effects of MP and determined under which conditions MI was most effective [13]. They defined five conditions of interest: 1) type of task, 2) retention interval, 3) experience level of trainees, 4) length of practice and 5) type of control group. The results of their meta-analysis showed a positive effect of MI when the following criteria were met: examination mainly of the cognitive aspects of the task performance, short retention interval, participants being novices to the task, and the MI session being about 20 minutes or shorter. They reported a non-significant trend for larger effects of MI compared with a non-treatment group and with an equivalent control treatment group.

### Imagery models and frameworks in the sports literature

In the sports psychology literature, six imagery models and frameworks were reviewed by Guillot and Collet [26]. The models included a four-component scheme originally designed by Martin *et al*., who described how MI influences cognitive, affective and behavioural outcomes [20]. The six-stage model from Munroe *et al*. was also evaluated, including the well known 'W' questions (where, when and why do athletes use MI, and what do they imagine?) [28]. This qualitative method includes the type (visual, kinaesthetic) and perspective (internal, external) of MI. MacIntyre and Moran extended the framework of Munroe *et al*. by adding the question: 'How should MI be executed and used by athletes?' [29], and they described a multimodal model that includes definition, outcome and importance of MI. Holmes and Collins introduced the PETTLEP framework, building on findings in functional neuroscientific research literature and experience in sport psychology [30]. PETTLEP aims to facilitate designing MI interventions for athletes, and comprises seven components (physical, environment, task, timing, learning, emotion and perspective). These components describe the physical position of the individual, the environment that has to be imagined, the task involved, the timing or duration of the imagery, the learning or changes involved during imagery, the emotions that are associated with the task, and imagery perspective. By contrast, the three-step model described by Watt *et al*. focused on MI ability and two image-generation approaches: 1) vividness, control, duration, ease, and speed; and 2) visual sensory methods [31]. The recent framework proposed by Guillot and Collet aimed to combine key components from previously described models. Their Motor Imagery Integrative Model in Sport (MIIMS) includes four MI outcomes: 1) motor learning and performance; 2) motivation, self-confidence and anxiety; 3) strategies and problem-solving; and 4) injury rehabilitation. The scheme aimed to combine different imagery types (visual, kinaesthetic, olfactory, tactile and auditory) to create a complete mental version of the movement [26].

### Motor imagery in medicine

MI research from sports psychology has been used in medicine, particularly in neurological rehabilitation [11-13]. Literature reviews have evaluated the overall beneficial effect of MI [5,9,11], but none has described the MITS elements or temporal parameters. In this review, we analysed the MITS elements and temporal parameters that have been successfully used in different disciplines: Education, Medicine, Music, Psychology and Sports (in this review, we use the term 'Sports' for all studies that include athletes as participants and the term 'Psychology' for all studies including healthy participants who are not athletes).

## Methods

### Search terms and search strategy

Search terms were identified by a previous search of databases (including PubMed) and internet search engines (including Google and Google Scholar). Additionally, each searched database was checked for pre-defined MeSH terms, and where available, these terms were integrated into the search strategy. The following terms were used: 'mental imagery', 'mental practice', 'mental rehearsal', 'mental movements', 'eidetic imagery', 'visual imagery', 'guided imagery', 'motor imagery' and 'mental training'. The Appendix provides the complete search strategy for Scopus. The Scopus search strategy was adapted to individual databases and trial registers to account for specific vocabulary and syntax rules. No restrictions were made regarding year of publication, study design or age of the study population.

### Study identification

Table 1 provides an overview of all databases, trial and dissertation registers, and conference proceedings searched. Database retrievals were imported into a reference management software package (EndNote; Thomson Reuters, Carlsbad, CA, USA). In total, 21,739 references were retrieved in February 2007. The literature search was repeated in June 2010 for references published between spring 2007 and 2010 in the largest databases of each discipline: the Education Resources Information Center (ERIC), Scopus, Répertoire International de Littérature Musicale (RILM), PsycINFO and SPORTDiscus. This search resulted in 5,741 additional references.

**Table 1:** Overview of searched databases, trial and dissertation registers and conference proceedings, and the number of references found

Number	Discipline	Database	Searched time period	References found, n
1	Education	Academic Search Premier	1975 to Feb 2007	1040

2	Medicine	AMED	1985 to Feb 2007	623

3	Education	ASSIA	1987 to March 2007	353
4		AEI	1979 to Feb 2007	84
5		BEI	1975 to Feb 2007	18

6	Medicine	BNI	1985 to Feb 2007	54
7		CINAHL	1982 to Feb 2007	1606
8		Cochrane Library	1948/1995 to march 2007	363
9		Digital dissertations	1930 to March 2007	30
10		DIMDI	1967 to March 2007	130

11	Sports	EMAERALD	1965 to March 2007	134

12	Education	ERIC	1966 to Feb 2007	795

13	Medicine	GMS meetings	2002 to March 2007	1

14		ISI Proceedings	1990 to March 2007	241

15	Music	JSTOR	1665 (1800) to Feb 2007	200

16	Psychology	PsycINFO	1887 to Feb 2007	4588

17	Music	RILM	1967 to March 2007	180

18	Medicine	Scopus	1996 to Feb 2007	2550

19	Sports	SPORTD*iscus*	1800 to Feb 2007	4023

20	Sports	SPORLIT, SPOFOR, SPORMED	1974 to Jan 2007	589

21	Medicine	ClinicalTrials.gov	1997 to March 2007	12
22		ISRCTN	1998 March 2007	2
23		National Research Register	2000 to March 2007	16
24		Web of Science	1970 to March 2007	2837
25		Zetoc	1993 to March 2007	1270

Total				21,739

AMED = Allied and Complementary Medicine, ASSIA = Applied Social Sciences Index and Abstracts, AEI = Australian Education International, BEI = British Education Index, BNI = British Nursing Index, CINAHL = Cumulative Index to Nursing and Allied Health Literature, DIMDI = German Institute for Medical Documentation and Information, ERIC = Educational Resources Information Center, GMS = General Medical Services, ISI = Web of Knowledge, JSTOR = Journal STORage, RILM = Répertoire international de Littérature Musicale, SPORLIT = Sporlit(eratur), SPORFOR = Sporfor(schung), SPORMED = Spormed(ia), ISRCTN = International Standard Randomised Controlled Trial Number

### Study selection criteria

The references were selected for review inclusion based on the following criteria:

• Any design of quantitative intervention studies with a focus on imagining movements.

• Studies that included healthy volunteers, students, children, professionals, athletes or patients from any discipline.

• Study intervention that focused on motor skill, performance or strength improvement.

The following exclusion criteria were used:

• Mental practice not related to movements (audition, odour, any kind of visual imagery with static pictures).

• Mental practice based on a computer-animated technique (virtual reality).

• Mental practice used during a functional magnetic resonance imaging (fMRI) session.

• Mental practice carried out during hypnosis or psychotherapy (guided imagery, eidetic imagery).

• Mental practice used as mental rotation or diagnostic tool.

• Suggested frameworks without participant evidence or experience.

• Publication language other than English or German.

### Selection process

**Figure 1:**
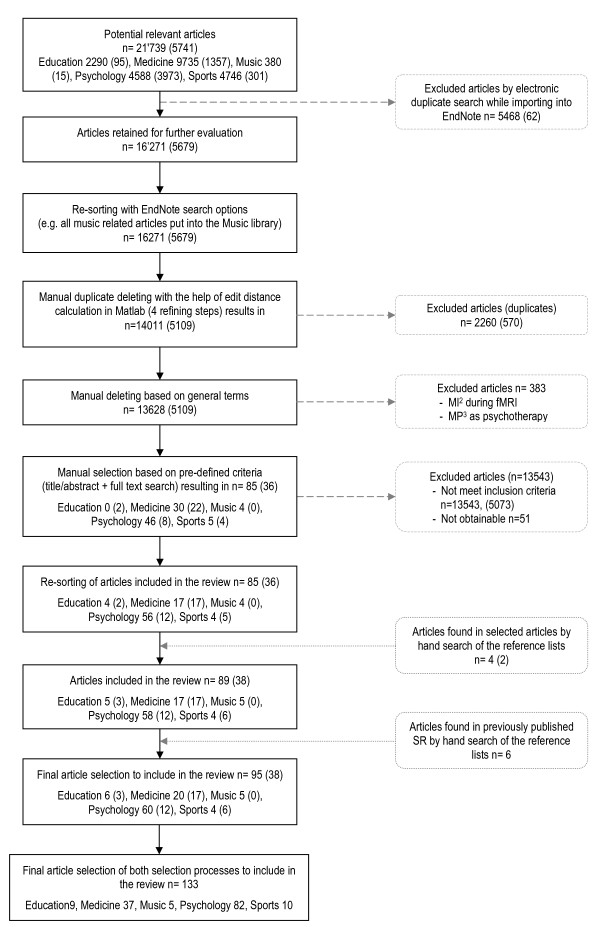
**The literature selection process**. Numbers in brackets indicate references retrieved from the search in June 2010. MI = motor imagery; MP = mental practice.

During the manual selection process (Figure 1), articles were evaluated based on title, abstract or keywords. Two of the authors (CS and RH) reviewed the articles; CS evaluated all references, and RH evaluated a randomly selected proportion (20%) of the initial number of references selected from each discipline. Full texts were ordered if no decision could be made based on the available information. If one reviewer could not reach a decision for a reference, this reference was discussed by both reviewers, and if both reviewers had not been able to agree on a decision (which was not the case in this investigation) a third reviewer (JB) would have been consulted.

Owing to the large number of references, EndNote search options were used to eliminate studies based on the exclusion criteria.

To confirm the selection congruency between both independent reviewers, the inter-rater congruency was calculated. Reviewer agreement ranged between 78% and 100% (average 96%) for the five disciplines. Because some studies reported more than one MI intervention, the total exceeded the number of included studies. Each MI intervention was subsequently analysed as an independent investigation.

### Data extraction

**Figure 2:**
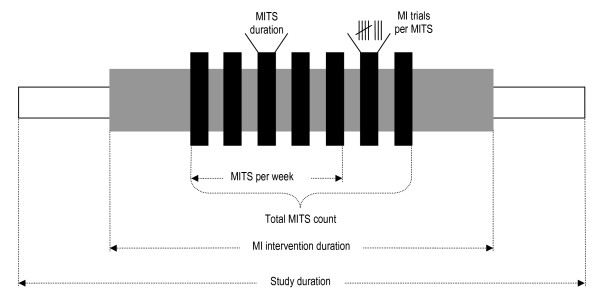
**Overview of extracted and calculated temporal parameters**. MI = motor imagery; MITS = motor imagery training session; total MI time: = (total MITS count) × (MITS duration).

Information on study methods, MITS elements and temporal parameters were extracted by three researches (RB, AS, CS) and checked for accuracy (CS). Table 2 summarises all extracted information. Figure 2 illustrates the temporal parameters and the MITS terminology.

**Table 2:** Overview of extracted MITS^a ^elements

Number	MITS element	MITS element description and categories	PETTLEP category	Dominant category found in successful MI^b ^interventions
1	Position	Describes the position of the individual during MI practice as *task*-*specific *or *not task*-*specific*.	Physical	Task-specific

2	Location	Describes the location of MITS as task-specific or *not task*-*specific*.	Environment	Task-specific

3	Focus	Each task consists of different parts. Focus of the intervention classifies the main focus of task-related activities that had to be imagined: *motor*, *cognitive or strength*.	Task	Motor-focused activities

4	Order	Describes temporal order of MI and PP^c ^trials. MI trials could have been performed *before*, *between*, *after *or *simultaneously *with PP.	Timing	MI after PP
5	Integration	Describes whether MI practice has been *added *to PP or *embedded*^*d *^into PP.		Added

6	MI instructions medium	MI instructions can be provided differently through one or more media types. Media type was scored as *acoustic*, *written *or *visual*. More than one media type could be assigned.	Learning	Acoustic
7	Instruction mode	In addition to the instruction medium, the mode was classified as *live *or *pre*-*recorded *(for example, using tape or video).		Live
8	Supervision	MITS could have been *supervised *or *not supervised *by an instructor present during the session.		Supervised
9	Directedness	MITS could have been *directed*^*d *^or *non-directed *when stepwise guidance was present or not.		Non-directed
10	Instruction type	The description of MI instructions varied. Instructions could cover *detailed *descriptions for each part of the task that had to be imagined, *simple keywords*, or *coarse *(*broad) *overall MI instructions.		Detailed
11	Instruction individuali-sation	MI instructions could have been individualised to the participant's problems with the task that had to be imagined (*tailored*), or could have been the same for each participant (*standardised*).		Standardised
12	Familiari-sation	Describe whether study participants had received an MI *familiarisation session before *the MI intervention began.		No familiarisation
13	Change	Indicated whether modification of content, duration or dosage of the MI training occurred, to facilitate the learning process during the MI intervention period.		

14	MI session	MITS could have been classified as *group *sessions with more than one person participating in a MITS or as *individual *sessions with one participant only.	Emotion	Individual
15	Eyes	During the MI, the participant's eyes could have been *closed *or *open*. In some interventions, participants started with one condition and changed to the other after one or several MITS.		Closed

16	Perspective	During the MI, participants could have imagined the task from an *internal *or *external *perspective. In some interventions, participants started with one condition and changed to the other after one or several MITS.	Perspective	Internal
17	Mode	During the MI, participants could have used a *kinaesthetic *or *visual *mode. In some MI interventions, participants started with one condition and changed to the other during one or after several MITS.		Kinaesthetic

^a^Motor imagery training session.

^b^Motor imagery.

^c^Physical practice.

^d^Used in MI interventions with no change or negative results, and differing from successful MI interventions.

### Study quality rating

Two rating lists were used because studies with different quantitative designs were included. The Physiotherapy Evidence Database (PEDro) list was used to evaluate randomised controlled trials (RCTs) (maximum of ten points) and non-RCTs (maximum of eight points) [32]. For case series or single cases experimental designs, the 11-point Single Case Experimental Design (SCED) scale was used [33]. All studies were rated by CS based on detailed rating guidelines. Studies received one point for each fulfilled methodological criterion on the respective rating list. The higher the achieved score, the better the study quality.

### Data analysis

Raw information was extracted into Excel (Microsoft Corp., Redmond, WA, USA). After coding and classification, MI intervention data was imported into statistical analysis software packages (SPSS versions 16 and 17 (SPSS Inc., Chicago, IL, USA), MATLAB version 2009b (The MathWorks Inc., Natick, MA, USA)) for frequency analyses, frequency and mean comparison tests and visualisation. MI intervention data was not pooled or analysed for significant differences because of the variability in experimental settings and missing information in MI intervention descriptions. The heterogeneity between MI interventions was also present in standard deviations of temporal parameters. All MI interventions were classified into two categories: positive change (129 MI interventions, 91.5%), and no or negative change from pre- to post-test (12 MI interventions, 8.5%). MITS elements and temporal parameters of studies with positive change were summarised under the term 'average positive MI intervention' and used for comparison in three analyses.

First, trend analyses were performed to identify MITS elements for MI interventions with positive results versus no changes or negative results. Further analyses aimed to identify changing trends in MITS element frequencies in MI interventions with positive results for five different disciplines, integration approaches, MI training focus, session type, age and gender groups and MI intervention modifications. Secondly, the χ^2 ^was used to test for significant differences between actual and expected observation frequencies for each MITS element. The tests were performed if 20% of the expected frequency showed an amount of 5 or more [34]. Thirdly, for temporal parameters, normal distribution was tested using the Kolmogorov-Smirnov test, and variance homogeneity was confirmed by the Levene test. Depending on the test results, group means were compared using Student *t*-test or Mann-Whitney *U*-test. The tests were used if at least five observations were available to estimate the statistic. For all temporal parameters, group means were compared against the average positive MI intervention.

For all statistical tests p ≤ 0.05 was considered significant.

## Results

**Figure 3:**
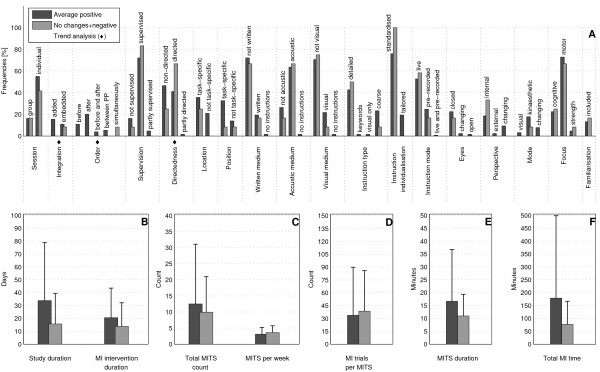
**Comparison of motor imagery (MI) interventions with positive results versus no change or negative results**. The figure shows the frequencies of motor imagery training session (MITS) elements and temporal parameter statistics for this analysis. Categories of MITS elements added up to 100% if an element was reported for all interventions considered in this analysis. For temporal parameters, bars show mean and positive SD. ♦ = Indicate changing trend of MITS element frequencies (see main text for detailed description); ο, Δ, ∇ = indicate significant results of the statistical tests against the average positive MI intervention.

**Figure 4:**
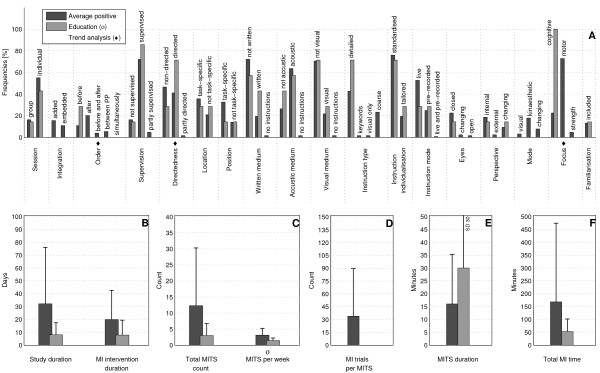
**Comparison of average positive motor imagery (MI) intervention versus discipline-specific MI interventions in Education**. The figure shows the frequencies of motor imagery training session (MITS) and temporal parameter statistics for successful interventions. Categories of MITS elements added up to 100% if an element was reported for all interventions considered in this analysis.. For temporal parameters, bars show mean and positive SD. ♦ = Indicate changing trend of MITS element frequencies (see main text for detailed description); ο, Δ, ∇ = indicate significant results of the statistical tests against the average positive MI intervention.

**Figure 5:**
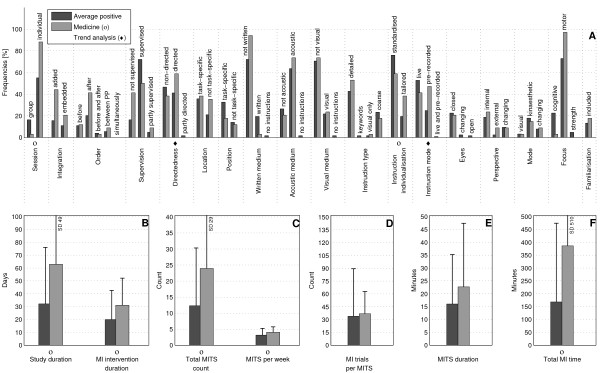
**Comparison of average positive motor imagery (MI) intervention versus discipline-specific MI interventions in Medicine**. The figure shows the frequencies of motor imagery training session (MITS) and temporal parameter statistics for successful interventions. Categories of MITS elements add to 100%, if an element was reported for all interventions considered in this analysis. For temporal parameters, bars show mean and positive standard deviation (SD). ♦ = Indicate changing trend of MITS element frequencies (see main text for detailed description); ο, Δ, ∇ = indicate significant results of the statistical tests against the average positive MI intervention.

**Figure 6:**
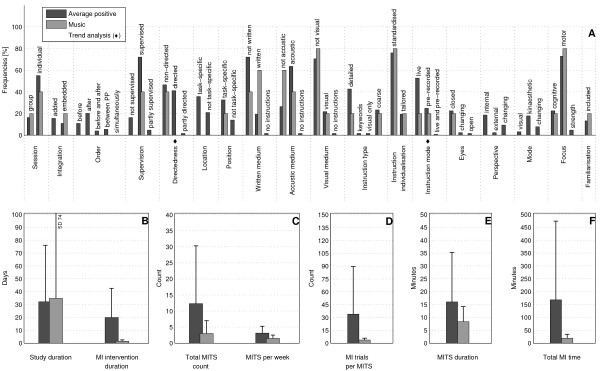
**Comparison of average positive motor imagery (MI) intervention versus discipline-specific MI interventions in Music**. The figure shows the frequencies of motor imagery training session (MITS) and temporal parameter statistics for successful interventions. Categories of MITS elements add to 100%, if an element was reported for all interventions considered in this analysis. For temporal parameters, bars show mean and positive standard deviation (SD).♦ = Indicate changing trend of MITS element frequencies (see main text for detailed description); ο, Δ, ∇ = indicate significant results of the statistical tests against the average positive MI intervention.

**Figure 7:**
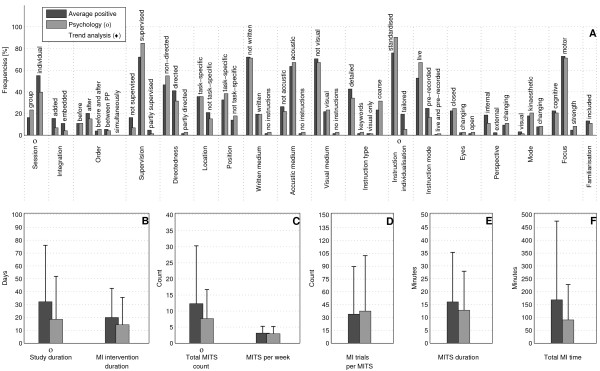
**Comparison of average positive motor imagery (MI) intervention versus discipline-specific MI interventions in Psychology**. The figure shows the frequencies of motor imagery training session (MITS) and temporal parameter statistics for successful interventions. Categories of MITS elements add to 100%, if an element was reported for all interventions considered in this analysis. For temporal parameters, bars show mean and positive standard deviation (SD).♦ = Indicate changing trend of MITS element frequencies (see main text for detailed description); ο, Δ, ∇ = indicate significant results of the statistical tests against the average positive MI intervention.

**Figure 8:**
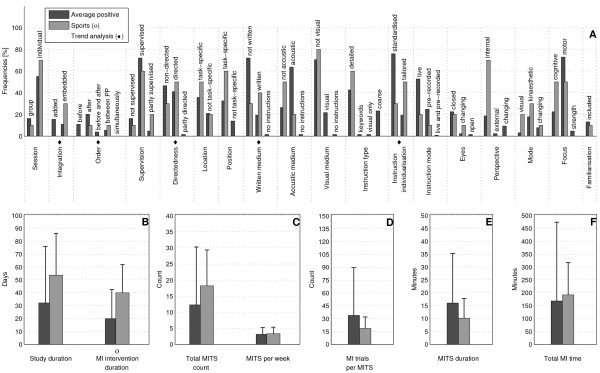
**Comparison of average positive motor imagery (MI) intervention versus discipline-specific MI interventions in Sports**. The figure shows the frequencies of motor imagery training session (MITS) and temporal parameter statistics for successful interventions. Categories of MITS elements add to 100%, if an element was reported for all interventions considered in this analysis. For temporal parameters, bars show mean and positive standard deviation (SD).♦ = Indicate changing trend of MITS element frequencies (see main text for detailed description); ο, Δ, ∇ = indicate significant results of the statistical tests against the average positive MI intervention.

**Figure 9:**
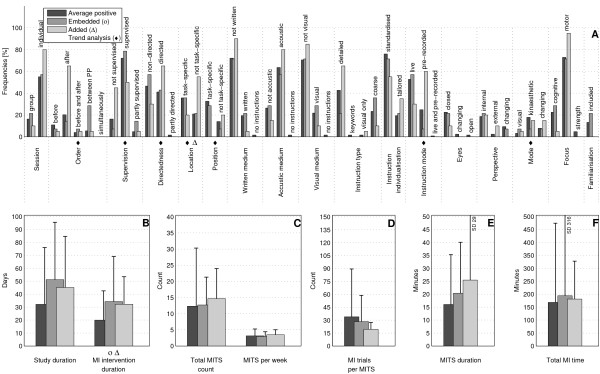
**Comparison of average positive motor imagery (MI) intervention versus MI integration approaches**. The figure shows the frequencies of motor imagery training session (MITS) and temporal parameter statistics for successful interventions. Categories of MITS elements add to 100%, if an element was reported for all interventions considered in this analysis. For temporal parameters, bars show mean and positive standard deviation (SD). ♦ = Indicate changing trend of MITS element frequencies (see main text for detailed description); ο, Δ, ∇ = indicate significant results of the statistical tests against the average positive MI intervention.

**Figure 10:**
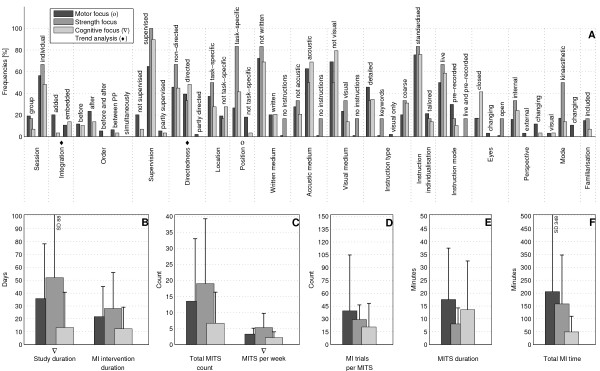
**Comparison of motor imagery (MI) interventions with different MI focus**. The figure shows the frequencies of motor imagery training session (MITS) and temporal parameter statistics for successful interventions. Categories of MITS elements add to 100%, if an element was reported for all interventions considered in this analysis. For temporal parameters, bars show mean and positive standard deviation (SD). The average positive MI intervention mirrored the frequency analysis of interventions with motor-related focus and is thus not shown.♦ = Indicate changing trend of MITS element frequencies (see main text for detailed description); ο, Δ, ∇ = indicate significant results of the statistical tests against the average positive MI intervention.

**Figure 11:**
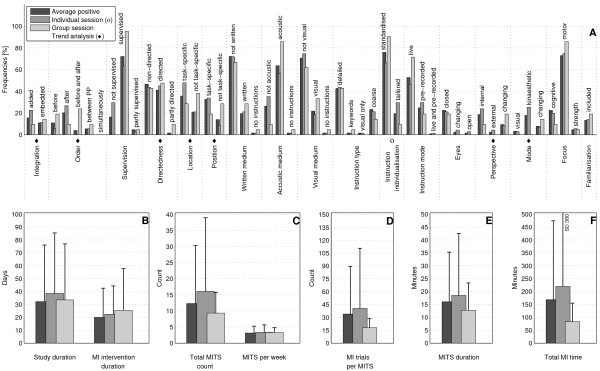
**Comparison of average positive motor imagery (MI) intervention versus different MI session types**. The figure shows the frequencies of motor imagery training session (MITS) and temporal parameter statistics for successful interventions. Categories of MITS elements add to 100%, if an element was reported for all interventions considered in this analysis. For temporal parameters, bars show mean and positive standard deviation (SD). ♦ = Indicate changing trend of MITS element frequencies (see main text for detailed description); ο, Δ, ∇ = indicate significant results of the statistical tests against the average positive MI intervention.

**Figure 12:**
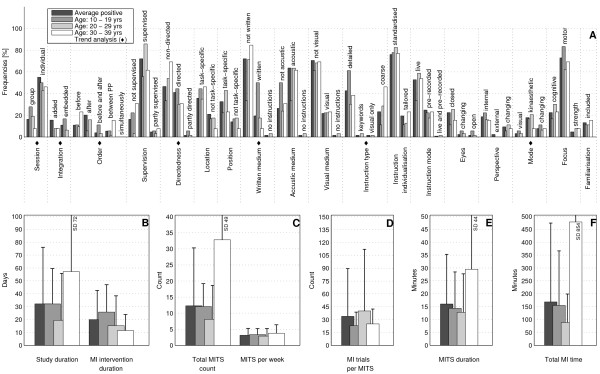
**Comparison of average positive motor imagery (MI) intervention versus different age groups (1)**. The figure shows the frequencies of motor imagery training session (MITS) and temporal parameter statistics for successful interventions. Categories of MITS elements add to 100%, if an element was reported for all interventions considered in this analysis. For temporal parameters, bars show mean and positive standard deviation (SD).♦ = Indicate changing trend of MITS element frequencies (see main text for detailed description); ο, Δ, ∇ = indicate significant results of the statistical tests against the average positive MI intervention.

**Figure 13:**
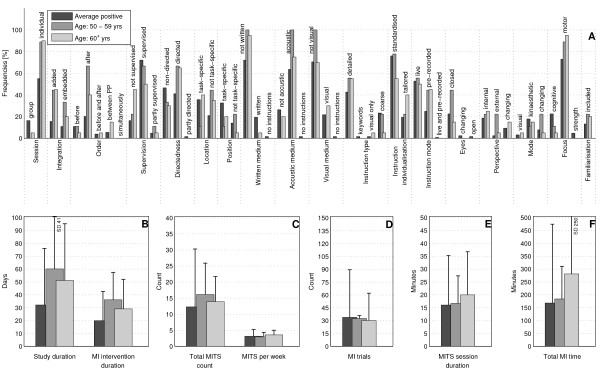
**Comparison of average positive motor imagery (MI) intervention versus different age groups (2)**. The figure shows the frequencies of motor imagery training session (MITS) and temporal parameter statistics for successful interventions. Categories of MITS elements add to 100%, if an element was reported for all interventions considered in this analysis. For temporal parameters, bars show mean and positive standard deviation (SD). ♦ = Indicate changing trend of MITS element frequencies (see main text for detailed description); ο, Δ, ∇ = indicate significant results of the statistical tests against the average positive MI intervention.

**Figure 14:**
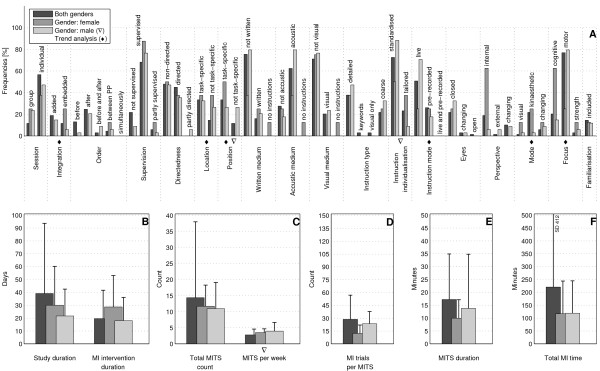
**Comparison of motor imagery (MI) interventions with regard to gender**. The figure shows the frequencies of motor imagery training session (MITS) and temporal parameter statistics for successful interventions. Categories of MITS elements add to 100%, if an element was reported for all interventions considered in this analysis. For temporal parameters, bars show mean and positive standard deviation (SD). The average positive MI intervention mirrored the frequency analysis of interventions with both genders and is thus not shown. ♦ = Indicate changing trend of MITS element frequencies (see main text for detailed description); ο, Δ, ∇ = indicate significant results of the statistical tests against the average positive MI intervention.

**Figure 15:**
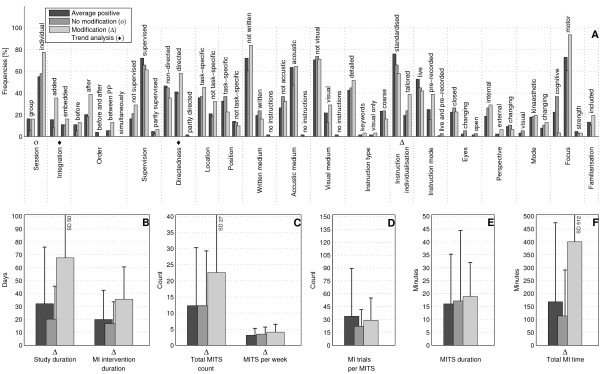
**Comparison of average positive motor imagery (MI) intervention versus intervention modifications (content, duration, dosage)**. The figure shows the frequencies of motor imagery training session (MITS) and temporal parameter statistics for successful interventions. Categories of MITS elements add to 100%, if an element was reported for all interventions considered in this analysis. For temporal parameters, bars show mean and positive standard deviation (SD). ♦ = Indicate changing trend of MITS element frequencies (see main text for detailed description); ο, Δ, ∇ = indicate significant results of the statistical tests against the average positive MI intervention.

The bar charts of plot A (Figure 3; Figure 4; Figure 5; Figure 6; Figure 7; Figure 8; Figure 9; Figure 10; Figure 11; Figure 12; Figure 13; Figure 14; Figure 15) show the frequencies of MI interventions that reported details on MITS elements. For each MITS element, one or more categories were considered; for example, for the MITS element 'session', the categories 'group' and 'individual' were analysed. The categories of MITS elements added up to 100% if an element was reported for all MI interventions in the respective analysis. Relevant trends in MITS elements, as reported in the text below, were marked in plots A to F in the same figures. For temporal parameters, bars show mean and positive SD.

### Study characteristics

In total, 133 studies were included in the analysis, reporting 141 MI interventions in five disciplines: Education (9 Interventions), Medicine (37), Music (5), Psychology (79) and Sports (11). For the studies published between 1941 and 2010, there were peaks in 1989/1990 (8 publications), in 2004 (18) and 2007 to 2009 (38). In Medicine, MI publications first appeared in 2000, with a steady increase until 2010. These studies originated from Europe, Australia/New Zealand, the Americas, Asia, and the Middle East.

The study designs comprised 91 randomised controlled trials (RCTs), 22 controlled clinical trials (CCTs), 15 case series (CSs) and 13 single-case research designs (SCRDs). Study quality was rated on a 10-point scale for RCTs (4 to 9), an 8-point scale for CCTs (3 to 6), an 11-point scale for CSs (4 to 11), and on an 11-point scale for SCRDs (7 to 10).

On average, RCTs and CCTs scored 6 on the 10-point PEDro scale, whereas CSs and SCRDs scored 6 and 8, respectively, on the 11-point SCED scale (on both, higher scores indicate better quality). Examples of MI instructions were available for 29 MI interventions, and changes in MI content during the MI intervention period were reported in 31 MI interventions. An overview of essential study characteristics is provided for each discipline separately (Table 3, Table 4, Table 5, Table 6 and Table 7).

**Table 3:** Overview of extracted descriptive study data for the discipline Education

Reference	First author	Year	Country^a^	Language	Study duration, days	Intervention duration, days	Study design	Study groups	Number of participants	Participants	Gender	Age, years	Body part	Training task	Focus	Measurement events	Results^b^		Quality rating
																		
																	Relative change	Absolute change	
[93]	Bucher, L	1993	USA	English	999	999	RCT	3	108	Nursing students	NSt	Range 19 to 21	Upper limb	Remove sterile gloves	M	1 (post-test)	↗	↗	5/10

[94]	Doheny, MO	1993	USA	English	1	1	RCT	4	95	Nursing students	Both	Mean = 21, range 18 to 40	Upper limb	Intramuscular injection	M	2 (pre-post test)	→	NSt	5/10

[95]	Immenroth, M	2005	DE	English	2	1	RCT	3	98	Surgeons	NSt	Mean ± SD = 32 ± 4	Upper limb	Laparoscopic cholecystectomy	M	2 (pre-post test)	↗	↗	9/10

[96]	Komesu	2009	USA	English	999	1	RCT	2	68	Surgeons	NSt	NSt	Upper limb	Surgical cystoscopy	C	1 (post-test)	↗	↗	8/10

[97]	Sanders, CW	2004	USA	English	21	21	RCT	3	65	Medical students	NSt	Students	Upper limb	Basic surgical procedures	M	2 (pre-post test)	→	↘	7/10

[98]	Sanders	2008	USA	English	15	2	RCT	2	64	Medical students	NSt	NSt	Upper limb	Basic surgical procedures	C	3 (post-tests, FU)	↗	↗	9/10

[99]	Stig, LC	1989	UK	English	1	1	RCT	2	35	Chiropractic students	Both	Mean = 23, range 19 to 40	Upper limb	Chiropractic adjustment skill	M	2 (pre-post test)	→	↗	6/10

[100]	Welk, A	2007	DE	English	999	999	RCT	2	41	Dentistry students	Both	Mean = 23	Upper limb	Preparation of tooth crown	C	2 (pre-post test)	↗	↗	8/10

[60]	Wright, CJ	2008	UK	English	999	28	RCT	2	56	Students	Both	University Students	Upper limb	Measuring blood pressure, antiseptic dressing task	C	2 (pre-post test)	↗	↗	8/10

^**a**^Countries: AU = Australia, BE = Belgium, BR = Brazil, CA = Canada, DE = Germany, ES = Spain, FR = France, G = German, GR = Greece, HK = Hong Kong, IL = Israel, IR = Iran, IT = Italy, KR = South Korea, NL = The Netherlands, NZ = New Zealand, PT = Portugal, SE = Sweden, UK = United Kingdom, USA = United States of America.

^b^Two elements were used to describe the study results: relative and absolute change: relative change evaluates the MI group results versus results of other study groups, while absolute change indicates the change of the MI group from pre- to post-test. ↗, →, ↘ = indicate trends of the study results from pre- to post-test (↗ positive change, → - no change, ↘ - negative change, ≈ = no precise numbers of measurement events stated in the publication)

Abbreviations: BL = Baseline, C = cognitive, CG = control group, CRPS1 = complex regional pain syndrome type 1, int. = Intervention, M = motor, N/A = not applicable, NK, not known;,NSt = not stated, S = strength

**Table 4:** Overview of extracted descriptive study data for the discipline Medicine

Reference	First author	Year	Country	Language	Study duration, days	Intervention duration, days	Study design	Study groups	Number of participants	Participants	Gender	Age, years	Body part	Training task	Focus	Measurement events	Results		Quality rating
																	Relative change	Absolute change	
[84]	Bovend'Eerdt, TJH	2009	UK	E	999	56	RCT	2	11	Stroke, MS, TBI	Both	Mean ± SD = 50 ± 14	Whole body	Muscle stretching	M	2 (pre-post-test)	→	→	7/10

[58]	Bovend'Eerdt, TJH	2010	UK	E	126	35	RCT	2	30	Stroke, TBI, MS	Both	Mean ± SD = 50 ± 14	Lower limb	ADL tasks	M	3 (pre-post-test, FU)	→	↗	8/10

[101]	Cramer, SC	2007	USA	E	9	7	CS	N/A	20	SCI	NSt	Mean ± SD = 31 ± 4	Tongue, foot	Tapping	M	2 (pre-post-test)	N/A	↗	9/11

[102]	Crosbie, J	2004	UK	E	35	14	SCRD	N/A	10	Stroke	Both	Range 45 to 81	Upper limb	Reaching, grasping	M	10 (BL, during int., FU)	N/A	↗	10/11

[59]	Dickstein, R	2004	IL	E	42	42	SCRD	N/A	1	Stroke	Male	69	Lower limb	Walking	M	5 (BL, midterm, post-test, FU)	N/A	↗	9/11

[103]	Dijkerman, R	2004	UK	E	28	28	CCT	3	20	Stroke	Both	Mean ± SD = 64 ± 9	Upper limb	Reaching, grasping	M	2 (pre-post-test)	↗	↗	5/10

[104]	Dunsky, A	2006	IL	E	77	42	SCRD	N/A	4	Stroke	Male	Mean = 58, (64, 57, 63, 47)	Lower limb	Walking	M	5 (BL, midterm, post-test, FU)	N/A	↗	9/11

[105]	Dunsky, A	2008	IL	E	77	21	CS	N/A	17	Stroke	Both	Mean = 58	Lower limb	Walking	M	6 (BL, pre-test, during int., post-test, FU)	N/A	↗	11/11

[38]	Guillot, A	2009	FR	E	14	999	RCT	2	14	hand burn	Both	Mean ± SD = 47 ± 14, range 27 to 74	Upper limb	Wrist + finger movements	M	≈ 6 (pre-test, during int., post-test)	↗	↗	7/10

[106]	Gustin, SM	2008	AU	E	15	7	CS	N/A	15	SCI	Male	Mean = 47, range 26 to 67	Lower limb	Plantarflexion, dorsiflexion	M	2 (pre-post-test)	N/A	↘	8/11

[107]	Hewett, T	2007	USA	E	56	42	SCRD	N/A	5	Stroke	Both	Mean = 53 ± 5, range 38 to 76	Upper limb	Reaching, grasping	M	2 (pre-post-test)	N/A	↗	7/11

[108]	Jackson, PL	2004	CA	E	35	21	SCRD	N/A	1	Hemorrhage- related lesion	Male	38	Lower limb	Foot serial response time task	M	2 (pre-post-test)	N/A	↗	8/11

[109]	Liu, K	2004	HK	E	21	21	RCT	2	46	Stroke	Both	Mean = 72	Whole body	ADL tasks	M	3 (pre-post-test, FU)	↗	↗	7/10

[110]	Liu, K	2004	HK	E	49	14	SCRD	N/A	2	Stroke	Both	65, 66	Whole body	ADL tasks	M	3 (pre-post-test, FU)	N/A	↗	7/11

[111]	Liu, KPY	2009	HK	E	999	2	RCT	2	33	Stroke	Both	Mean = 70 ± 8	Whole body	ADL tasks	M	2 (pre-post-test)	↗	↗	8/10

[62]	Malouin, F	2004	CA	E	2	1	CS	N/A	12	Stroke	Both	Mean = 53 ± 12	Lower limb	Symmetrical load standing up + sitting down	M	3 (pre-post-test, FU)	N/A	↗	9/11

[63]	Malouin, F	2009	CA	E	42	21	RCT	3	12	Stroke	Both	Mean = 61 ± 8, range 53 to 75	Lower limb	Symmetrical load standing up + sitting down	M	3 (pre-post-test, FU)	↗	↗	8/10

[112]	McCarthy, M	2002	UK	E	999	999	SCRD	N/A	2	CVA, TBI	Male	64, 36		Neglect	M	3 (pre-test, during int., post-test)	N/A	↗	9/11

[113]	Moseley, GL	2004	AU	E	210	14	RCT	2	13	CRPS1 after wrist fracture	Both	Mean ± SD = 37 ± 15	Upper limb	Hand + finger movements	M	5 (pre-test, during int., post-test, FU)	↗	↗	7/10

[114]	Moseley, GL	2005	AU	E	126	14	RCT	3	20	CRPS1 after wrist fracture	Both	Mean = 34 ± 8	Upper limb	Hand + finger movements	M	5 (pre-test, during int., post-test, FU)	↗	↗	7/10

[47]	Moseley, GL	2006	AU	E	84	14	RCT	2	51	Phantom limb, CRPS1	Both	37	Upper limb	Hand + finger movements	M	3 (pre-post-test, FU)	↗	↗	7/10

[86]	Moseley, GL	2008	Western Europe + AU	E	1	1	CCT	2	37	CRPS1, no-CRPS1 pain	Both	Mean ± SD = 41 ± 14	Upper limb	Hand + finger movements	M	2 (pre-post-test)	→	↘	5/10

[115]	Mueller, K	2007	DE	E	98	28	RCT	3	17	Stroke	Both	Mean ± SD = 62 ± 10	Upper limb	Finger+hand movements	M	8 (BL, during int., post-test, FU)	→	↗	6/10

[116]	Page, SJ	2000	USA	E	28	28	RCT	2	16	Stroke	Male	Mean = 63	Upper limb	Weightbearing + functional task	M	2 (pre-post-test)	↗	↗	7/10

[65]	Page, SJ	2001	USA	E	56	42	SCRD	N/A	1	Stroke	Male	56	Upper limb	Whole arm movements	M	3 (BL, post-test)	N/A	↗	7/11

[64]	Page, SJ	2001	USA	E	56	42	RCT	2	13	Stroke	Both	Mean ± SD = 65, range 64 to 79	Upper limb	Whole arm movements	M	3 (BL, post-test)	↗	↗	7/10

[117]	Page, SJ	2005	USA	E	56	42	RCT	2	11	Stroke	Both	Mean = 62 ± 5, range 53 to 71	Upper limb	Hand ADL tasks	M	3 (BL, post-test)	↗	↗	8/10

[118]	Page, SJ	2007	USA	E	999	72	CS	N/A	4	Stroke	Both	Mean = 63, range 49 to 73	Upper limb	Hand ADL tasks	M	2 (pre-post-test)	N/A	↗	10/11

[119]	Page, SJ	2007	USA	E	63	42	RCT	2	32	Stroke	NSt	Mean ± SD = 60 ± 14	Upper limb	Hand ADL tasks	M	3 (BL, post-test)	↗	↗	8/10

[120]	Page, SJ	2009	USA	E	91	70	CS	N/A	10	Stroke	Both	Mean = 57 ± 12, range 37 to 69	Upper limb	Whole arm ADL tasks	M	3 (BL, post-test)	N/A	↗	9/11

[121]	Page, SJ	2009	USA	E	168	70	RCT	2	10	Stroke	Both	Mean ± SD = 61 ± 3, range 48 to 79	Upper limb	Whole arm ADL tasks	M	4 (BL, post-test, FU)	↗	↗	8/10

[122]	Riccio, I	2010	IT	E	42	21	RCT	2	36	Stroke	Both	Mean = 60 ± 12	Upper limb	Whole arm ADL tasks	M	3 (pre-test, first + second study part)	↗	↗	8/10

[123]	Simmons, L	2008	UK	E	999	10	CS	CS	10	Stroke	Both	Mean = 68 ± 14	Upper limb	Whole arm movements	M	3 (pre-post-test, FU)	N/A	↗	7/11

[66]	Stenekes, MW	2009	NL	E	84	42	RCT	2	25	Surgery for carpal tunnel syndrome	Both	Mean ± SD = 34 ± 11	Upper limb	Passive bending + straightening wrist + fingers	M	3 (pre-post-test, FU)	↗	↗	7/10

[124]	Stevens, JA	2003	USA	E	128	28	SCRD	N/A	2	Stroke	Both	76; 63	Upper limb	Wrist movements, object manipulation	M	4 (pre-test, during int., post-test, FU)	N/A	↗	7/11

[125]	Tamir, R	2007	IL	E	84	84	RCT	2	23	Parkinson disease	Both	Mean ± SD = 67 ± 10	Whole body	ADL tasks	M	2 (pre-post-test)	↗	↗	7/10

[126]	Yoo, EY	2006	KR	E	10	999	SCRD	N/A	3	Stroke	Male	Mean = 57, (46, 70, 56)	Lower limb	Symmetrical weightbearing	M	21 (BL, during int., post-test, FU)	N/A	↗	9/11

^**a**^Countries: AU = Australia, BE = Belgium, BR = Brazil, CA = Canada, DE = Germany, ES = Spain, FR = France, G = German, GR = Greece, HK = Hong Kong, IL = Israel, IR = Iran, IT = Italy, KR = South Korea, NL = The Netherlands, NZ = New Zealand, PT = Portugal, SE = Sweden, UK = United Kingdom, USA = United States of America.

^b^Two elements were used to describe the study results: relative and absolute change: relative change evaluates the MI group results versus results of other study groups, while absolute change indicates the change of the MI group from pre- to post-test. ↗, →, ↘ = indicate trends of the study results from pre- to post-test (↗ positive change, → - no change, ↘ - negative change, ≈ = no precise numbers of measurement events stated in the publication)

Abbreviations: BL = Baseline, C = cognitive, CG = control group, CRPS1 = complex regional pain syndrome type 1, int. = Intervention, M = motor, MS = multiple sclerosis; N/A = not applicable, NK, not known;,NSt = not stated, S = strength, SCI = spinal cord injury, TBI = traumatic brain injury

**Table 5:** Overview of extracted descriptive study data for the discipline Music

Reference	First author	Year	Country	Language	Study duration, days	Intervention duration, days	Study design	Study groups	Number of participants	Participants	Gender	Age, years	Body part	Training task	Focus	Measurement events	Results		Study rating
																	Relative change	Absolute change	
[127]	Coffman, DD	1990	USA	E	1	1	CCT	8	2	Musicians	Both	Mean = 23, range 18 to 58	Upper limb	Piano-playing performance	M	2 (pre-post-test)	↗	↗	5/10

[128]	Ross, SL	1985	USA	E	1	1	RCT	5	51	Trombonists	Both	Mean = 22, range 18 to 29	Upper limb	Trombone-playing performance	M	2 (pre-post-test)	↗	↗	7/10

[129]	Rubin-Rabson, G	1941	USA	E	168	999	CCT	3	13	Piano teachers	NSt	range 21 to 25	Upper limb	Piano-playing performance, memorising new études	M	3 (during int., post-test)	↗	↗	4/10

[130]	Sonnen-schein, I	1990	DE	G	3	3	CS	N/A	20	Piano players	Both	Mean = 33, range 14 to 51	Upper limb	Piano-playing performance	M	2 (pre-post-test)	N/A	↗	4/11

[131]	Theiler, T	1995	USA	E	1	1	CCT	4	14	Music students: guitar majors, voice majors	NSt.	Range 19 to 29	Upper limb	Guitar-playing + vocal performances	M	2 (pre-post-test)	↗	↗	5/10

**Table 6:** Overview of extracted descriptive study data for the discipline Psychology

Reference	First author	Year	Country	Language	Study duration, days	Intervention duration, days	Study design	Study groups	Number of participants	Participants	Gender	Age, years	Body part	Training task	Focus	Measurement events	Results		Study rating
																	Relative change	Absolute change	
[132]	Allami, N	2008	FR	E	1	1	RCT	5	25	Students	NSt	Mean = 29, range 20 to 37	Upper limb	Grasping task	M	2 (pre-post-test)	↗	↗	7/10

[133]	Alves, J	1999	PT	E	28	28	CCT	4	64	Pupils	Both	Range 15 to 17	Whole body	Volleyball service	M	2 (pre-post-test)	→	↗	3/10

[134]	Andre, C	1986	USA	E	5	5	CCT	3	66	Students	Male	Mean = 21 ± 3	Whole body	Frisbee disc gold putting/throwing	M	2 (pre-post-test)	→	↗	4/10

[135]	Chevalier, H	1986	CA/FR	E	1	1	RCT	5	30	Students	Both	Undergraduate students	Upper limb	Moving computer mouse	C	1 (post-test only)	↗	↗	6/10

[136]	Clark, LV	1960	USA	E	28	21	CCT	2	144	Pupils	Male	High-school pupils	Whole body	Pacific coast one-hand foul shot	M	2 (pre-post-test)	→	↗	5/10

[137]	Clegg, BC	2004	USA	E	1	1	SCRD	N/A	28	Older adults, students	Both	Old: mean = 74, range 62 to 88; young: mean = 22, range 18 to 26	Upper limb	Movement with stylus	M	1 (post-test only)	N/A	→	8/11

[138]	Corbin, CB	1967	USA	E	28	21	RCT	3	30	Pupils	Male	High-school pupils	Whole body	Wand-juggling skill	M	2× BL, post-test, FU 1 day	↘	↗	5/10

[139]	Cornwall, MW	1991	USA	E	4	4	RCT	2	24	Females	Female	Mean = 23, range 21 to 25	Lower limb	Strength of quadriceps muscle	S	2 (pre-post-test)	↗	↗	6/10

[140]	Decety, J	1991	USA	E	1	1	RCT	2	20	Students	Both	Mean = 23 ± 2	Lower limb	Walking on beam	M	5 (during int., post-test)	↗	↗	6/10

[41]	Etnier, J	1996	USA	E	1	1	RCT	9	153	Students	Both	Mean ± SD = 23 ± 4	Whole body	Basketball shooting	M	3 (pre-test, during int., post-test)	↗	↗	6/10

[141]	Gassner, K	2007	DE	G	999	21	RCT	2	36	Students	Both	Mean = 24	Lower limb	Walking with knee prosthesis	M	2 (pre-post-test)	↗	↗	7/10

[142]	Gordon, S	1994	AU	E	21	21	RCT	3	64	High-school pupils	NSt	High-school pupils	Whole body	Cricket outswing	M	6 (pre-test, during int., post-test)	→	↗	6/10

[143]	Gray, SW	1990	USA	E	21	14.0	RCT	2	24	Males	Male	Mean = 22, range 18 to 26	Whole body	Forehand and backhand racquetball skills	M	2 (pre-post-test)	↗	↗	6/10

[144]	Hellwing, W	1976	DE	G	14	14	CCT	2	72	Pupils	Male	Mean = 12, range 11 to 13	Whole body	Fosbury flop	M	1 (post-test)	→	↗	4/10

[145]	Hemayattalab, R	2009	IR	E	38	24	RCT	5	40	Mentally retarded children	NSt	Mean = 14, range 12 to 15	Whole body	Basketball free throw	M	3 (pre-post-test, FU)	↗	↗	6/10

[146]	Herrero, J	2004	ES	E	7	7	CCT	2	27	Students	Female	Mean ± SD = 20 ± 0.1	Upper limb	Bench-press	S	2 (pre-post-test)	↗	↗	6/10

[56]	Isaac, AR	1992	NZ	E	126	126	CCT	2	70	Students	NSt	NSt	Whole body	Three trampoline skills	M	6 (after 1, 6, 7, 12, 13, 18 weeks)	→	→	6/10

[147]	Jaehme, W	1978	DE	G	21	14	RCT	3	48	Pupils	Male	Mean = 16	Whole body	Crawl swimming	M	2 (pre-post-test)	↗	↗	5/10

[42]	Jarus, T	2000	IL	E	1	1	RCT	2	89	Children, adults	Both	Children: mean ± SD = 10 ± 1; adults: 28 ± 5; older adults: 67 ± 2	Upper limb	Two-arm coordination task	C	6 (during int., FU)	↗	↗	6/10

[148]	Jones, JG	1965	AU	E	14	14	RCT	2	71	Students	Male	Students	Whole body	Hock-swing upstart	M	2 (during int., post-test)	↗	↗	7/10

[149]	Kelsey, IB	1961	CA	E	22	2	RCT	3	36	Students	Male	University students	Trunk, lower limb	Endurance abdominal + thigh-flexor muscles	M	2 (pre-post-test)	↗	↗	7/10

[150]	Kohl, RM	1980	USA	E	1	1	RCT	3	60	Students	NSt	Mean = 21	Upper limb	Pursuit rotor task	C	28 (during int., post-test)	→	↗	5/10

[150]	Kohl, RM	1980	USA	E	1	1	RCT	3	60	Students	Male	Mean = 20	Upper limb	Pursuit rotor task	C	36 (during int., post-test)	→	↗	5/10

[150]	Kohl, RM	1980	USA	E	1	1	RCT	6	108	Pupils	Male	Mean = 17	Upper limb	Pursuit rotor task	C	NSt	↘	↗	5/10

[151]	Kornspan, AS	2004	USA	E	5	4	RCT	4	40	Students	Both	Mean = 20	Whole body	Golf putting	M	3 (pre-post-tes)t	→	↗	6/10

[152]	Kremer, P	2009	AU	E	1	1	RCT	4	209	Students	Both	Mean ± SD = 21 ± 3	Whole body	Dart throwing with non-preferred hand	M	2 (pre-post-test)	→	↗	7/10

[153]	Krigolson, O	2006	CA	E	1	1	CCT	6	42	Healthy participants	NSt	Range 18 to 32	Lower limb	Walking along walkway	M	20 (during int., post-test)	→	↗	4/10

[57]	Lejeune, M	1994	BE	E	7	4	CCT	4	40	University students + staff	Both	Mean = 22, range 19 to 27	Whole body	Counterattack forehand and backhand (table tennis)	M	3 (Pre-post-test, FU)	↗	↗	5/10

[154]	Linden, CA	1989	USA	E	14	8	RCT	2	23	Healthy participants	Female	Mean = 79, range 67 to 90	Whole body	Walking balance, equilibrium reactions	M	3 (pre-test, during int., post-test)	→	→	8/10

[45]	Lutz, R	2001	USA	E	1	1	RCT	5	120	Students	Both	Undergraduate students	Whole body	Golf putting	M	2 (pre-post-test)	↗	↗	7/10

[155]	Maring, JR	1990	USA	E	1	1	RCT	2	26	University students + staff	Both	Mean = 30; range 22 to 40	Upper limb	Tossing a ping-pong ball to target	M	2 (pre-post-test)	↗	↗	6/10

[67]	Martin, KA	1995	CA	E	6	6	RCT	3	39	Students	Both	Mean ± SD = 27 ± 6	Whole body	Golf putting	M	2 (pre-post-test)	↗	↗	6/10

[156]	McAleney, P	1990	USA	E	999	21	RCT	2	20	Students	Both	Mean = 19, range 18 to 20	Whole body	Tennis shooting skills	M	2 (pre-post-test)	→	↗	7/10

[157]	Minas, SC	1978	UK	E	1	1	RCT	4	32	Students	Both	Undergraduate students	Whole body	Throwing performance	M	2 (pre-post-test)	→	↗	6/10

[68]	O, J	2008	CA	E	999	1	RCT	5	97	Healthy students	Both	Mean ± SD = 18 ± 2	Whole body	Dribbling a soccer ball	M	2 (pre-post-test)	↗	↗	7/10

[158]	Papaxanthis, PC	2002	FR	E	1	1	RCT	2	16	Students	Both	Mean = 21, range 19 to 23	Lower + upper limb	Walking + writing task	M	5 (during int., post-test)	→	↗	6/10

[159]	Phipps, SJ	1969	USA	E	21	21	RCT	2	72	Students	Male	University students	Whole body	Hock swing, jump-foot, soccer hitch kick	M	2 (pre-post-test)	↗	↗	6/10

[160]	Ranganathan, VK	2004	USA	E	231	84	RCT	4	30	Healthy participants	Both	Mean ± SD = 30 ± 5	Upper limb	Muscle strength of little finger abduction, elbow flexion	S	18 (BL, during int., FU)	→	↗	6/10

[35]	Rapp, G	1973	DE	G	14	9	RCT	3	58	Pre-school children	Both	Mean = 6	Whole body	Skipping	M	2 (pre-post-test)	→	↗	7/10

[161]	Rawlings, E	1972	USA	E	11	10	RCT	3	24	Students	Female	Undergraduate students	Upper limb	Rotary pursuit tracking	C	10 (pre-test, during int., post-test)	→	↗	6/10

[161]	RawlingsE	1972	USA	E	10	9	RCT	2	20	Students	Male	Students	Upper limb	Rotary pursuit tracking	C	10 (pre-test, during int., post-test)	→	↗	5/10

[69]	Reiser, M	2005	DE	G	28	28	RCT	3	34	Students	Both	Mean ± SD = 24 ± 2, range 20 to 27	Upper limb	Bench-press	S	4 (pre-test, during int., post-test)	↘	↗	6/10

[83]	Rodrigues, EC	2010	BR	E	1	1	CS	N/A	18	Students	Both	Mean = 26, range 19 to 33	Lower limb	Plantar flexion	M	2 (pre-post-test)	↗	↗	9/11

[162]	Ryan, E	1981	USA	E	1	1	RCT	3	39	Students	Male	Undergraduate students	Upper limb + whole body	'Dial-a-maze' pattern, stabilometer performance	M	2 (pre-post-test)	↘	↗	5/10

[163]	Ryan, E	1982	USA	E	1	1	RCT	6	80	Traffic officers	Male	Mean = 36, range 23 to 57	Whole body	Stabilometer performance	M	4 (pre-test, during int., post-test)	↗	↗	7/10

[164]	Shackell, EM	2007	CA	E	21	10	RCT	3	30	Students	Male	Mean ± SD = 20 ± 2	Lower limb	Strength-training of hip flexor muscle	S	2 (pre-post-test)	↗	↗	7/10

[165]	Sidaway, B	2005	USA	E	28	28	RCT	3	24	Students	Both	Mean = 23, range 19 to 26	Lower limb	Ankle dorsiflexor torque	M	2 (pre-post-test)	→	↗	7/10

[166]	Singer, RN	1970	USA	E	35	28	RCT	5	65	Students	Female	College students	Upper limb	Learning a pursuit rotor task	C	2 (pre-post-test)	→	↗	5/10

[167]	Smith, LE	1962	USA	E	1	1	CCT	6	60	Students	Male	Mean = 20, range 17 to 27	Upper limb	Hand-eye coordination task; punchboard learning task	M	2 (pre-post-test)	→	↗	4/10

[72]	Smith, D	2001	UK	E	21	21	RCT	2	27	Students	Both	Mean ± SD = 20 ± 3	Whole body	Landing hockey penalty	M	2 (pre-post-test)	↗	↗	7/10

[71]	Smith, D	2004	UK	E	49	49	RCT	3	19	University students + staff	Male	Mean ± SD = 30 ± 8	Upper limb	Strength of abductor digiti minimi	S	2 (pre-post-test)	→	↗	7/10

[71]	Smith, D	2004	UK	E	1	1	RCT	4	24	University students + staff	Both	Mean ± SD = 29 ± 8	Upper limb	Barrier knock-down task	C	2 (pre-post-test)	→	↗	7/10

[168]	Smyth, MM	1975	UK	E	1	1	RCT	7	70	Students	Both	Undergraduate and postgraduate students	Upper limb	Mirror drawing of a star	C	2 (pre-post-test)	↘	↗	5/10

[168]	Smyth, MM	1975	UK	E	1	1	RCT	7	71	Students	Both	Undergraduate and postgraduate students	Upper limb	Pursuit rotor task	C	1 (post-test)	↗	↗	5/10

[169]	Start, KB	1960	AU	E	9	9	CS	N/A	35	Pupils	Male	12	Whole body	Basketball throw	M	2 (pre-post-test)	N/A	↗	6/11

[170]	Start, KB	1964	AU	E	7	6	CS	N/A	21	Students	Male	Mean = 20, range 18 to 21	Whole body	Single leg upstart on high-bar	M	1 (post-test)	N/A	↗	7/11

[87]	Start, KB	1964	AU	E	7	6	CS	N/A	44	Students	Male	Mean = 19, range 18 to 25	Whole body	Single leg upstart on high-bar	M	1 (post-test)	N/A	→	7/11

[88]	Start, KB	1964	AU	E	14	6	CS	N/A	32	Students	Male	Mean = 20, range 18 to 21	Whole body	Single leg upstart on high-bar	M	1 (post-test)	N/A	→	6/11

[171]	Stebbins, RJ	1968	USA	E	42	21	RCT	5	93	Students	Male	College students	Whole body	Throwing ball into target	M	8-18 (pre-test, during int., post-test)	→	↗	5/10

[172]	Surburg, PR	1968	USA	E	63	56	CCT	7	183	Students	Male	Junior college students	Whole body	Tennis forehand drive	M	2 (pre-post-test)	→	↗	5/10

[36]	Taktek, K	2004	CA	E	1	1	CCT	4	64	Children	Both	Mean = 9, range 8 to 10	Upper limb	Pushing play vehicle	C	2 (pre-post-test)	→	↗	4/10

[73]	Toussaint, L	2010	FR	E	3	2	RCT	8	80	Students	Both	Mean ± SD = 23 ± 3	Lower limb	Knee joint angles	M	3 (pre-post-test, FU)	→	↗	6/10

[173]	Tunney, N	2006	USA	E	2	2	RCT	2	19	Older adults	Both	Mean = 76, range 66 to 89	Lower limb	Walking with quad cane + climbing four stairs	M	2 (pre-post-test)	→	↗	7/10

[174]	Twining, W	1949	USA	E	22	20	RCT	3	36	Students	Male	College students	Whole body	Throwing rings at target	M	2 (pre-post-test)	-1	↗	4/10

[175]	van Gyn, GH	1990	CA	E	42	42	RCT	4	40	Students	Both	Undergraduate students	Lower limb	Power + sprint performance on ergometer	M	2 (pre-post-test)	→	↗	6/10

[176]	Vandell, RA	1943	USA	E	20	18	RCT	3	36	Pupils and college students	Male	Junior, senior high-school and college students	Whole body	Throwing darts at target, basketball free throws	M	2 (pre-post-test)	→	↗	5/10

[37]	Vergeer, I	2006	UK	E	28	28	RCT	3	36	University staff	Both	Mean ± SD = 41 ± 10	Lower limb	Flexibility around hip joint	M	2 (pre-post-test)	→	↗	7/10

[74]	Wakefield, CJ	2009	UK	E	999	28	RCT	4	32	Students	Female	University students	Whole body	Netball shooting performance	C	2 (pre-post-test)	↗	↗	7/10

[75]	White, KD	1979	AU	E	9	8	CCT	4	24	High-school pupils + university students	NSt	Mean = 19, range 13 to 27	Whole body	Action-reaction swimming start	M	2 (pre-post-test)	→	↗	4/10

[177]	Whiteley, G	1966	UK	E	84	84	CCT	4	88	Pupils	Male	Mean = 11	Whole body	Neck spring, head spring, short-arm overswing	M	1 (post-test)	↗	↗	3/10

[76]	Williams, JG	2004	UK	E	21	21	RCT	3	24	Undergraduate students	Both	Mean ± SD = 21 ± 2	Lower limb	Rom hip flexion	M	6 (pre-test, during int., post-test, FU)	↗	↗	7/10

[178]	Wohldamm, EL	2007	USA	E	84	2	CCT	4	80	Students	NSt	Undergraduate and postgraduate students	Upper limb	Number typing task	M	2 (post-test)	→	↗	4/10

[178]	Wohldamm, EL	2007	USA	E	1	1	CCT	4	108	Students	NSt	Undergraduate and postgraduate students	Upper limb	Number typing task	M	3 (pre-test, FU)	→	↗	5/10

[179]	Woolfolk, RL	1985	USA	E	1	1	RCT	6	48	Students	Male	Undergraduate college students	Whole body	Putt golf balls into cup	M	2 (pre-post-test)	→	↗	7/10

[180]	Woolfolk, RL	1985	USA	E	7	6	RCT	3	30	Students	Both	College students	Whole body	Golf backswing and putting stroke	M	2 (pre-post test)	↗	↗	5/10

[77]	Wright, CJ	2009	UK	E	999	42	RCT	5	50	Students	NSt	Mean ± SD = 21 ± 4	Upper limb	Biceps curl task	S	2 (pre-post-test)	↗	↗	7/10

[78]	Yaguez, L	1998	DE	E	1	1	CCT	2	58	Volunteers	Both	Mean ± SD = 35 ± 11, range 22 to 73	Upper limb	Ideogram drawing	C	3 (pre-test, during int., post-test)	→	↗	6/10

[78]	Yaguez, L	1998	DE	G	1	1	CCT	2	52	Volunteers	Both	Mean = 30, range 22 to 49	Upper limb	Connecting circles	C	3 (pre-test, during int., post-test)	↗	↗	6/10

[181]	Zecker, SG	1982	USA	E	1	1	RCT	4	40	Students	Both	Undergraduate college students	Whole body	Tossing beanbag to target	M	2 (pre-post-test)	↗	↗	5/10

**Table 7:** Overview of extracted descriptive study data for the discipline Sports

Reference	First author	Year	Country	Language	Study duration, days	Intervention duration, days	Study design	Study groups	Number of participants	Participants	Gender	Age, years	Body part	Training task	Focus	Measurement events	Results		Study rating
																	Relative change	Absolute change	
[182]	Casby, A	1998	UK	E	84	46	SCRD	N/A	4	Expert swimmers	Both	Mean = 16, range 16 to 17	Whole body	Freestyle swimming turn	M	50 (pre-test, during int., post-test)	N/A	→	8/11

[54]	Grouios, G	1992	GR	E	14	7	RCT	5	100	Top level athletes	Male	Mean = 22, range 18 to 25	Upper limb	Pushing button	C	2 (pre-post test)	↗	↗	7/10

[183]	Guillot, A	2009	FR	E	56	42	CS	N/A	10	Basketball players	Female	Mean = 23	Whole body	Three strategic basketball tactics	C	2 (pre-post test)	N/A	↗	8/11

[55]	Guillot, A	2010	FR	E	999	35	RCT	2	21	Synchronised swimmers	Female	Mean = 15, SD 2	Whole body	Stretching exercises	M	2 (pre-post test)	↗	↗	7/10

[184]	Olsson, CJ	2008	SE	E	999	42	RCT	2	19	High-jump athletes	Both	Mean = 19 ± 3, range 16 to 29	Whole body	High jump	M	2 (pre-post test)	↗	↗	6/10

[79]	Robin, N	2007	FR	E	70	56	CCT	3	30	Tennis players	NSt	Mean = 19 ± 3	Whole body	Tennis service return	M	2 (pre-post test)	→	↗	6/10

[185]	Shambrook, CJ	1996	UK	E	84	84	SCRD	N/A	4	Basketball players	Female	Mean = 20, SD 2	Whole body	Basketball free throw	M	26 (pre-test, during int., post-test)	N/A	↗	9/11

[80]	Smith, D	2007	UK	E	999	42	RCT	4	48	University hockey players	Both	Mean = 20 ± 3	Whole body	Field hockey penalty flicks	C	2 (pre-post test)	↗	↗	7/10

[80]	Smith, D	2007	UK	E	999	42	RCT	4	40	Junior gymnasts	Female	Mean = 10 ± 2, range 7 to 14	Whole body	Full turning straight jump	C	2 (pre-post test)	→	↗	8/10

[81]	Smith, D	2008	UK	E	999	42	RCT	4	32	Golf players	Male	NSt	Whole body	Hitting golf ball out of bunker	M	2 (pre-post test)	↗	↗	7/10

[186]	Ziemainz, H	2003	DE	G	14	2	RCT	3	27	Triathletes	Both	Mean = 16, range 15 to 17	Whole body	Changing between triathlon-specific sports	M	3 (pre-post test, FU)	→	↗	5/10

### Comparison of MI interventions with positive results versus no change or negative results: how should a successful MI intervention be implemented?

The MITS elements for all MI interventions were compared (Figure 3A). Frequency analyses of MI interventions with positive results revealed a number of key MITS elements present in a successful intervention design: MI was performed in individual sessions and added after PP; MI sessions were supervised and not directed; locations of MITS and the position of the participants during MI were both task-specific; participants received acoustic and detailed MI instructions, which were mainly standardised and live; during MI practice, participants kept their eyes closed.; the perspective used during MI practice was chosen from an internal view combined with a kinaesthetic MI mode; and MI interventions were mainly investigated with motor-focused tasks.

Only 17 reports mentioned an MITS for familiarisation before the MI intervention began. The reporting rate of all MITS elements ranged between 26% for the description of closed or open eyes to 95% for MI instruction individualisation. The most frequently reported MITS elements in successful MI interventions are listed in Table 2.

MI interventions with no change or with negative results predominantly used directed MITS. If MI integration was reported, MITS were *embedded between *or performed *simultaneously *with physical trials. Owing to the lack of reporting, the ordering of MI and PP could not be identified in 90% of all MI interventions. Only two MI interventions mentioned an MITS for familiarisation before MI intervention began. For the subsequent analyses only successful MI interventions with positive results were considered.

MI interventions with positive results had almost twice the duration of MI interventions with no change or negative results: study duration (34 days), MI intervention duration (21 days), total MITS count (13), (the number 13 stands for the number of MITS in MI interventions with positive results)MITS duration (17 minutes) and total MI time (178 minutes). By contrast, MI interventions with no change or negative results had a larger number of MITS per week (3) and a larger number of MI trials per MITS (34).

### Comparison of positive MI interventions in five different disciplines: how do different disciplines use MI?

In the described analyses, only positive MI interventions were considered. The two disciplines with the youngest participants were Psychology (aged up to 9 years) and Sports (10 to 19 years). Most MI interventions were carried out with students (20 to 29 years) in Education, Psychology, Music, and Sports. Participants aged 50 and older were included only for Medicine and Psychology. Gender-specific investigations were carried out in Medicine, Psychology and Sports.

Detailed discipline-specific frequency analyses of MITS elements revealed the following differences (italics) from the average positive MITS.

For Education, participants (Figure 4A-F) performed MI predominantly *before *physical practice (PP) during *directed *MITS. Three MITS elements showed both categories: position of participants during MITS (task-specific and not task-specific), instruction mode (life and pre-recorded), and perspective (internal and external). MI content focused on *cognitive *task-related activities. MI mode was not reported. Regarding the temporal parameters in Education, the study and MI intervention duration and the total MI time were less than half of those in the average positive MI intervention, but the MITS duration was twice as long as in the average positive MITS. The number of MI trials per MITS was not reported.

MI interventions in Medicine (Figure 5A-F), the MI interventions predominantly used *directed *MITS with *pre-recorded *MI instructions. All temporal parameters had longer durations and total counts compared with the average positive MITS, especially for study and MITS duration, total MITS count and total MI time.

MI interventions in Music (Figure 6A-F) tended to be *embedded *into PP. MI instructions in Music were mainly *written*. Instruction mode and type allowed for more than one categorisation. Location of the MITS, and the MI perspective and mode used were not described. Almost all temporal parameters had lower durations and numbers than in the average positive MITS, particularly the number of MI trials per MITS, but the study duration was higher for MI interventions in Music.

MI interventions in Psycholog*y *(Figure 7A-F) most closely resembled the average positive MITS with similar distributions of MITS elements in instruction type and in MI perspective. MI interventions had the same number of MITS per week to the average positive MITS, but shorter MI intervention and MITS duration. Total MI time was half of that of the average positive MI intervention.

MI interventions in Sports (Figure 8A-F) reported *embedded *and *directed *MITS, [and *after *or *between *PP. Instructions during MITS were *tailored *and in *written *form. Study and MI intervention duration were almost twice as long as those in the average positive MI intervention. MI trials in a Sports MITS were only half of the number of the average positive MITS.

### Comparison of added and embedded MI integration approaches: does a specific set of MITS element for each method exist?

This analysis was performed in successful MI interventions, which included PP in the study design. Of the 34 retrieved MI interventions, 20 described an added and 14 an embedded MI training method (Figure 9A-F). There was a preference for added MITS to be *directed *using *pre-recorded *instructions. *Neither *the locations of MITS nor the position of the participants during MI practice were *task-specific*. Added MITS used a *kinaesthetic *or *mixed *MI mode. MI training embedded into PP tended to be *supervised*, and was implemented *between *physical trials of the same task. Most MI interventions did not report details on location and position.

The averages of the temporal parameters of both categories (*added *and *embedded*) differed from those of the average positive MI intervention; however, there was wide variation between MI interventions. The duration of the study MI intervention and MITS duration were longer for both categories than in the average positive MITS, but the number of MI trials per MITS was lower. MITS duration was longer in the added than in the embedded training methods.

### Comparison of MI interventions with different MI training focus: is MI particularly suited to one training focus?

Based on the primary focus of activities that were imagined, positive MI interventions were categorised into *motor-focused *(94), cognitive-focused (29) and strength-focused (6) activities. Compared with motor and cognitive-focused MI interventions, which were mainly published between the 1970s and 2010, the majority of strength-focused MI interventions were published in 1991 and in the period 2004 to 2009. Motor- and strength-focused MI interventions were often designed according to the average positive MITS (Figure 10A-F). Strength-focused MI interventions were investigated in healthy participants aged 20 to 39 years only. Motor-focused MI interventions had the highest number of MI trials per MITS and the longest MITS duration and total MI time.

Cognitive-focused MI interventions differed from the average positive MI intervention: there was a preference for MITS to be *embedded *and *directed*. Cognitive-focused MI interventions had shorter durations and lower numbers in all temporal parameters compared with motor-focused MI interventions.

### Comparison of MI interventions with different MI session types: do group sessions require a different design from individual ones?

This analysis could be performed for 37 positive MI interventions that reported details. In total, 21 MI interventions described MITS in group sessions, and 71 in individual sessions (Figure 11A-F).

Group MITS tended to be *directed *and *embedded *into PP, and included MI practice *before *and *after *PP. *Neither *the locations of the MITS nor the position of the participants during MI practice were *task-specific*. Both the MI perspective used during MI practice and the MI mode *changed*. Total MI time and number of MI trials per session were only half those of the average positive MI intervention.

For the individual sessions, we investigated two options: directed and non-directed MITS. Compared with the average positive MI intervention, individual sessions had larger values for many of the temporal parameters, particularly total MI time.

### Comparison of MI interventions with regard to participant age: did the implementation differ for particular age groups?

Participant age in successful MI interventions was classified into seven categories: up to 9 years (2 interventions), 10 to 19 years (18), 20 to 29 years (63), 30 to 39 years (13), 40 to 49 years (2), 50 to 59 years (9), and 60 and older (20). Two MI interventions did not mention the age of the participant and were thus not considered in this analysis.

There were only two MI interventions with participants aged up to 9 years, published in 1973 [35] and 2004 [36]. Both were studies in Psychology, which considered healthy children of both genders with an average age of 6 and 9 years, respectively, and were *supervised *with *acoustic *instructions. Rapp and Schoder described the MI intervention as a *non-directed *group session with *live *and *standardised *instructions. Children *closed *their eyes during MI as they imagined a motor-focused task [35]. No further details were provided. Taktek *et al*. designed the MI intervention as a *directed *session with *pre-recorded *instructions [36]. Participants used a *task-specific *position during MI, *closed *their eyes and used a *kinaesthetic *MI mode when imagining a *cognitive-focused *task. MI trials were preformed *before *PP trials using *standardised *and *detailed *instructions. Temporal parameters differed between both investigations.

The MI interventions (n = 18) with teenagers (10 to 19 years) were in the fields of Psychology and Sports. Investigations were designed as *directed individual *sessions. Where reported, MI was practiced either *before*, or *before *and *after *PP. Participants received their MI instructions in *written *form. Only the number of MI trials per MITS was less than that in the average positive MI intervention (Figure 12A-F).

The MI interventions (n = 63) with participants aged between 20 and 29 year*s *most closely resembled the average positive MI intervention. Deviations were observed in three temporal parameters: study duration and total MI time were two-thirds of those in the average positive MI intervention, and participants performed more MI trials per session than in the average positive MI intervention.

MI interventions (n = 13) with participants aged between 30 and 39 years were mainly designed as *added *MITS with MI practice before PP and *coarse *(*broad) *MI instructions. MI mode was reported in two MI interventions as *kinaesthetic *and *changing *mode, respectively. Four temporal parameters had twice the duration or frequency than in the average positive MI intervention: study duration, MITS duration, total MITS count and total MI time. The number of MI trials per session was lower than those of the average positive MI intervention.

Only two MI interventions could be classified in the age group 40 to 49 years [37,38]. The MI intervention described by Vergeer and Roberts was performed with healthy participants of both genders [37]. MITS elements resembled those of the average positive MI intervention, with *group *sessions and a multimodal approach for MI instructions (*written, visual and acoustic*). The second article, published by Guillot *et al*., described an MI intervention with participants (male and female) who had burns [38]. The authors used *added*, *individual*, *supervised *and *directed *MITS. Participant received *detailed*, *tailored *and *written *instructions.

The MI interventions (n = 9) with participants aged between 50 to 59 years were *directed *MITS (Figure 13A-F). *Neither *the locations of the MITS nor the position of the participants during MI practice were *task-specific*. *Internal *and *external *MI perspective options were offered. A *changing *MI mode was preferred. Temporal parameters had almost twice the duration of MI study and MI intervention than in the average positive MI intervention.

The MI interventions (n = 20) with participants aged 60 years and older were *directed *MITS, similar to the previous age group. There was no deviation in MITS elements from those of the average positive MI intervention. Temporal parameters had a longer study duration and total MI time.

### Comparison of MI interventions with regard to participant's gender: should gender-specific settings be considered for MITS implementation?

Gender-specific analyses of positive MI interventions showed the following distribution: sixty-nine MI interventions with participants of both genders, eight MI interventions with female participants only and thirty-four MI interventions with male participants only. Gender-specific investigations were carried out in Medicine, Psychology and Sports.

The MI interventions with participants of both genders (n = 69) were designed according to the average positive MI intervention. They had the longest study duration and total MI time and the largest number of MI trials per session compared with gender-specific MI interventions (Figure 14A-F).

The MI interventions (n = 8) with female participants only were primarily designed as *embedded *MITS with MI trials *between *PP trials. The locations of the MITS were both *task-specific *and *non-task-specific*. MI instructions were *live *or *pre-recorded*. MI interventions with female participants were investigated with mainly cognitive-focused tasks. The MITS duration and number of MI trials were lower than those of MI interventions with both genders and with male participants only; however, the duration of the MI intervention was longer.

The MI interventions (n = 34) with male participants used *task-specific *or *non-task-specific *positions, and the MI mode selected was the *changing *mode. Temporal parameters closely resembled those of the average positive MI intervention.

### Analyses based on change in content, duration and dosage of MI training: what MITS element variations have been investigated?

Successful MI interventions were categorised with regard to change in MI intervention during the total MI intervention period into change (n = 31) and no change (n = 38). Change in MI intervention could include three domains: MI dosage, MI content and MI time. MI interventions were excluded from the analysis if a change was not clearly described (n = 55) or if the categorisation was not applicable (n = 5) due to the study design.

Only minor differences were found between categories (Figure 15A-F). MI interventions with a change during the MI intervention period included *directed *MITS. Duration of study and MI intervention, total MITS count, and total MI time were almost twice those of the average positive MI intervention. MI interventions without a change during the MI intervention period were designed as *embedded *MITS with shorter study duration, lower number of MI trials and lower total MI time than in the average positive MI intervention.

## Discussion

### Summary of findings

A question frequently raised by clinicians is 'How should motor imagery be done?'. Our literature review aimed to answer this question and to describe which elements characterise successful MITS. It was not our intention to evaluate the effectiveness of MI or to compare effect sizes, as this has already been addressed in other literature reviews [1,6,11,13]. The results of the trend analyses revealed changes in the frequencies of the MITS elements, which represent important variations between MI interventions. In addition, the review identified differences between the studies with positive results and those with no changes or negative results. Thus, the trend analyses might help clinicians to implement MI interventions successfully. By contrast, the χ^2 ^test revealed general frequency distribution differences only, which were often caused by frequency variations and did not represent actual trend changes. Owing to limitations in the reporting rates, the χ^2 ^test for MITS elements and the group mean comparison tests for temporal parameters could not be applied in many cases. We expect that our trend analyses in combination with the statistical test would be able to serve as indicators for potential future research directions.

Our analyses considered the differences in specific disciplines (Education, Medicine, Music, Psychology, Sports), MI integration types (added/embedded), session types (individual/group), focus of the task (motor, cognitive, strength), age, gender groups (female, male, both ) and change in content, duration and dosage. From 141 MI interventions, data were extracted and analysed for 17 MITS elements based on the PETTLEP approach and 7 temporal parameters.

#### MI intervention outcome

The comparison of the MI interventions with positive results versus those with no change or negative results provided the basis for all subsequent analyses. An average positive MI intervention was derived comprising MITS elements and temporal parameters. Characteristics of the average positive MI intervention were seen in studies in Psychology, in interventions with motor-focused or strength-focused tasks in all disciplines, in interventions with participants aged 20 to 29 years old, and in interventions with both genders. Four MITS elements differed between the MI interventions with positive results and those with no change or negative results: order (embedded/simultaneous), directedness (directed), number of MITS per week (n = 3) and number of MI trials per MITS (n = 34). We hypothesise that several of these elements jointly inhibit positive results. Depending on the length of a MITS and the experience level of the participant, the most frequent number of MITS per week chosen in successful MI interventions was three.

Data analyses determined that the average MITS duration was 17 minutes, with 34 MI trials per MITS. Both of these temporal parameters were also retrieved in the review of Feltz and Landers, published in 1983 [1], which yielded similar values. Our results suggest that not more than two MI trials per minute per MITS might be performed.

MI interventions with no change or negative results were present in all four study designs (RCT, CCT, CS and SCRD), with a higher average quality score for SCRDs than for RCTs. Therefore, it cannot be concluded that a certain design leads to a negative outcome.

#### Discipline-specific intervention adaptation

The use of imagery originated in the field of Psychology, with investigations dating back to publications in 1880 and 1897 [39,40]. Presumably, MITS adaptations were necessary to direct each step of a surgical procedure in Education, to tailor imagery tasks to the needs of participants in Medicine, to use written instructions (musical notes) in Music, and to embed MI between PP trials as recovery breaks during an intensive training day in Sports.

In the current review, the positive MI interventions were mainly performed after PP. This result stands in contrast to the reported order of performing MI trials before PP in the meta-analysis of Feltz and Landers and the investigation of Etnier and Landers [1,41]. No overall conclusion on the reported order could be derived because of its dependency on the aim of the MI training, such as the learning of a new motor task, its adaptation, preparation for performance of a known motor task, achievement of peak performance, and memorisation of performance aspects.

Temporal parameters varied between disciplines. The longest study and MI intervention durations and the highest total number of MITS were seen in Medicine and Sports. Some of these variations could be explained by their very nature. For example, in Medicine, time to learn and perform the MI was required, reflecting system impairments, older age of the participants and chronic pain, whereas in Sports, MITS can be part of the daily training routine. The longest MITS duration could be found in Music, reflecting the length of the music pieces that were imagined. Medicine and Psychology had the highest numbers of MI trials per MITS. This supports the hypothesis that MI is effective in these fields when the imagined movement is short and simple (for example, one limb movement) to perform, with as many repetitions as possible during a short concentration period, as described above in the section on MI intervention outcome.

#### MI session type

The decision to implement MITS as group or individual sessions does not depend on the MI integration approach. Both group and individual sessions included added and embedded MITS. Both classifications were used in positive MI interventions during the entire publication period analysed. The MI intervention duration was longer for group MITS and shorter for individual MITS compared with the positive MITS. We hypothesised that the selection of session type was based rather on practical considerations than on scientific reasoning. Further research is needed to evaluate the influence of session type on the effectiveness of MI interventions.

#### Age groups

Most MI interventions were performed with healthy students and young adults aged 20 to 29 years old. Hence there is a need for MI techniques and investigation of their effectiveness in young children and middle-aged adults, for which only a few references were found. Jarus and Ratzon reported that children aged 9 years and older adults aged 65 and 70 years benefited more from the combination of MI and PP than did young adults aged 21 to 40 years [42]. The full potential of MI in younger and older participants has not yet been sufficiently investigated, as evidenced by the low number of MI investigations found in these age groups.

#### Gender effect

In the current review gender differences were found in the chosen MITS elements. The results obtained will add to the ongoing debate on gender-dependent MI intervention design. Is it believed that males are better imagers than females, because of the different brain area activation and inhibition [43]. The 'bottom-up neural strategy' found in the work of Butler *et al*. could be related to the visuospatial performance benefit of men, with larger improvements for men gained from a motor-focused MI intervention compared with women [43]. This hypothesis could have influenced the MI intervention design in studies with female participants, which used mainly cognitive-focused tasks. However, a questionnaire survey given to healthy participants aged 18 to 65 years [44] did not confirm a gender imbalance on imagery usage. Furthermore, Lutz *et al*. did not detect a gender effect among high- and low-skilled golfers in a putting task after MI [45], nor were gender differences found in an investigation with two widely used imagery questionnaires [46]. In the current review, the study imbalance for female to male participants is 1 to 4.25. Therefore, we hope our analyses will prompt researchers to further explore potential gender differences in, for example, MI ability.

### Methodological considerations

The only available MeSH term for searches was 'mental imagery', which must be considered as an umbrella term for various mental techniques. MI is one technique focusing on movements, which is important in rehabilitation medicine. Historically, other terms have been used for the same purpose in literature. Our literature search included various terms associated with imagery, yielding a large initial reference count.

Studies were included regardless of their study design, country and year of publication. This method enabled us to obtain a global view of the MI literature in different disciplines and of the MI approaches that were evaluated in different study designs. We used and adapted two widely accepted scales to evaluate all studies for their methodological quality.

The analysed studies primarily investigated the short-term effect of MI with a simple pre-/post-test design. The longest time period evaluated was a 6-month follow-up in an RCT by Moseley *et al*., in which significant improvements were seen in the MI treatment group compared with a control group [47].

Overall, data reporting in the selected articles was low, and the implications of this are highlighted by one of the least reported elements: imagery perspective. Depending on the chosen perspective (first or third person), different brain areas will be activated [48]. Publications on successful and non-successful athletes reported contradictory results for the imagery perspectives used [46,49,50]. Furthermore, Kim *et al*. investigated the exercise-related imagery perspective in middle-aged adults and, reported an internal:external perspective ratio of 1.8 [51]. Mulder *et al*. found a slightly better MI vividness in adults over 64 years when using the external MI perspective. The authors also mentioned that MI from an internal perspective is more important than MI from an external perspective in learning a motor skill [52,53]. Furthermore, they could detect a shift in perspective related to age, with younger people more likely to use the internal perspective and older people more likely to use the external perspective. Taking imagery perspective as an example, future research should detail MITS elements more carefully.

### Limitations and outlook

There were two important sources of possible information bias: firstly, 51 references were not obtainable, and secondly, our selected references included only 12 MI interventions with no change or negative results versus 129 MI interventions with positive results. We therefore hypothesised that MI interventions without positive results are rarely published. This hypothesis is further supported by our identification during the selection process of abstracts detailing preliminary results of MI interventions but no follow-up full article describing the whole MI intervention and its final results. Nevertheless, the aim of this review was to analyse MI interventions with positive results, and to identify discipline-specific MI interventions and fundamental intervention designs.

We found that the reporting standard of MI intervention had improved in recent years; however, investigations published before 2007 often lacked details on MITS elements, which resulted in missing data in the frequency analyses. Many investigations included more than one experimental or control group. In such cases, we focused our analyses focused on the experimental group with the largest change in measurement between pre- and post-intervention measurement.

The MI interventions were heterogeneous, which explained the large standard deviations in temporal parameters.

Task evaluation is complex and subjective, and to date, no standard classification exists. In our review we classified the investigated tasks based on their main focus: motor, cognitive or strength.

Before applying an MI intervention, it is essential to evaluate the MI ability of the participants to determine whether they are able to perform MI. Additionally, MI ability might change over an intervention period. In the current literature review, we found that assessments of MI ability had been used in thirty-six studies with positive results [36-38,54-83] and in five studies with no change or with negative results [84-88]. Heterogeneity between the MI ability assessments used, which were partially custom-designed for individual MI interventions, prevented direct comparison and relation to the study results in this review. We hope this will encourage researchers to use assessments of MI ability and to state participant scores in their research reports.

This review does not include MI interventions that were published after June 2010, because of the reference selection and analysis process. However, we briefly mention new articles currently under review in Medicine. Braun *et al*. embedded MI training into regular therapy in patients with stroke in nursing homes and in patients with Parkinson disease at different disease levels. In both investigations, embedded MI did not show a significant advantage compared with the control group receiving regular care [89,90]. These interventions may add information for analysing positive results versus no change or negative results.

The current review focused on MITS elements to improve motor function or learn a motor skill. Further reviews should consider the influence of MI on psychological factors, such as goal-setting [91], self-efficacy, motivation and mood [92], and working memory.

## Conclusion

This review covering five disciplines identified key MITS elements and temporal parameters of a successful MI intervention design. Successful design characteristics were dominant in the Psychology literature for all of the following: interventions using motor and strength-focused tasks, interventions with participants aged 20 to 29 years old, and interventions including both genders. Four MI elements were identified that differed between experiments with positive results and those with no change or negative results; however, success was not related to intervention study design. MI interventions in Education, Medicine, Music and Sports were adapted for different MITS elements and temporal parameters. No distinct characteristics were identified regarding the choice of group or individual sessions. Reports on MI interventions did not use consistent terminology, and often lacked details on MITS elements and temporal parameters. We hope this review will prompt researchers to a coherent usage of the MI term, which could facilitate subsequent meta-analyses.

## List of abbreviations

CCT: clinical controlled trial; CS: case series; fMRI = functional magnetic resonance imaging; MI: motor imagery; MITS: Motor Imagery Training Session; MP: mental practice; PP: physical practice; PETTLEP: physical, environment, timing, task, learning, emotion, perspective; PEDro: Physiotherapy Evidence Database; RCT = randomised controlled trial; SCRD: single-case research design

## Conflicts of interest

The authors declare that they have no competing interests.

## Authors' contributions

CS, BA, JB, UK and TE made substantial contributions to conception and design of the review. CS, RH, OAM, AS made substantial contributions to acquisition of data, and analysis and interpretation of data. CS, OAM, BA, JB, UK, TE were involved in drafting the manuscript and critically revising it. All authors have given final approval of the manuscript.

## Appendix

### Example search strategy

Search strategy Scopus database from 22 February 2007: (((TITLE-ABS-KEY("mental imagery")) OR (TITLE-ABS-KEY-AUTH("mental practice")) OR (TITLE-ABS-KEY-AUTH("mental rehearsal")) OR (TITLE-ABS-KEY-AUTH("mental movements")) OR (TITLE-ABS-KEY-AUTH("eidetic imagery")) OR (TITLE-ABS-KEY-AUTH("visual imagery")) OR (TITLE-ABS-KEY-AUTH("guided imagery")) OR (TITLE-ABS-KEY-AUTH("motor imagery")) OR (TITLE-ABS-KEY-AUTH("mental training"))) AND NOT (TITLE-ABS-KEY-AUTH("mental health"))) AND NOT (TITLE-ABS-KEY-AUTH("body image")) 2,556 references

## Pre-publication history

The pre-publication history for this paper can be accessed here:

http://www.biomedcentral.com/1741-7015/9/75/prepub
